# IKK Epsilon Deficiency Attenuates Angiotensin II-Induced Abdominal Aortic Aneurysm Formation in Mice by Inhibiting Inflammation, Oxidative Stress, and Apoptosis

**DOI:** 10.1155/2020/3602824

**Published:** 2020-01-22

**Authors:** Hao Chai, ZhongHao Tao, YongChao Qi, HaoYu Qi, Wen Chen, YueYue Xu, LeiYang Zhang, HongWei Chen, Xin Chen

**Affiliations:** Department of Thoracic and Cardiovascular Surgery, Nanjing First Hospital, Nanjing Medical University, No. 68 Changle Road, Nanjing 210006, China

## Abstract

Abdominal aortic aneurysm (AAA) is a vascular disorder that is considered a chronic inflammatory disease. However, the precise molecular mechanisms involved in AAA have not been fully elucidated. Recently, significant progress has been made in understanding the function and mechanism of action of inhibitor of kappa B kinase epsilon (IKK*ε*) in inflammatory and metabolic diseases. The angiotensin II- (Ang II-) induced or pharmacological inhibitors were established to test the effects of IKK*ε* on AAA in vivo. After mice were continuously stimulated with Ang II for 28 days, morphologically, we found that knockout of IKK*ε* reduced AAA formation and drastically reduced maximal diameter and severity. We also observed a decrease in elastin degradation and medial destruction, which were independent of systolic blood pressure or plasma cholesterol concentrations. Western blot analyses and immunohistochemical staining were carried out to measure IKK*ε* expression in AAA tissues and cell lines. AAA phenotype of mice was measured by ultrasound and biochemical indexes. In zymography, immunohistology staining, immunofluorescence staining, and reactive oxygen species (ROS) analysis, TUNEL assay was used to examine the effects of IKK*ε* on AAA progression in AAA mice. IKK*ε* deficiency significantly inhibited inflammatory macrophage infiltration, matrix metalloproteinase (MMP) activity, ROS production, and vascular smooth muscle cell (VSMC) apoptosis. We used primary mouse aortic VSMC isolated from apolipoprotein E (Apoe) ^−/−^ and Apoe^−/−^IKK*ε*^−/−^ mice. Mechanistically, IKK*ε* deficiency blunted the activation of the ERK1/2 pathway. The IKK*ε* inhibitor, amlexanox, has the same impact in AAA. Our results demonstrate a critical role of IKK*ε* in AAA formation induced by Ang II in Apoe^−/−^ mice. Targeting IKK*ε* may constitute a novel therapeutic strategy to prevent AAA progression.

## 1. Introduction

Abdominal aortic aneurysm (AAA) is a chronic inflammatory vascular disease in the elderly population. A permanent AAA is typically diagnosed when the aorta is more than 1.5-fold normal diameter [1]. Smoking, male sex, age (>60 years), hypertension, and family history are other possible risk factors for AAA formation [2]. Clinically, despite the rapid development of medical imaging technology and surgical interventions, the clinical treatment of AAA is currently limited to endovascular techniques or open surgery for aneurysms larger than 5.5 cm. Pharmacological treatments are lacking for the condition, and effective nonsurgical treatments to modify the natural history of AAA progression have not been validated [3].

Inhibitor of kappa B kinase epsilon (IKK*ε*) is an important member of the IKK family, and its overexpression leads to malignant transformation of human disease [4, 5]. Using mouse deficiency in IKK*ε* and apolipoprotein E (Apoe), we recently showed that IKK*ε* is a key player in the pathogenesis of the cardiovascular disease [6, 7]; deficiency of IKK*ε* has been suggested to have an anti-inflammatory effect and to inhibit malignant transformation [8, 9]. Expression of IKK*ε* was upregulated in AAA patients when compared to a normal group. The current study is aimed at investigating the role of IKK*ε* in response to angiotensin II (Ang II) and at elucidating its role in AAA formation. We used a mouse model of inflammatory AAA [10], in which chronic subcutaneous infusion of Ang II occurred in Apoe^−/−^ and IKK*ε*^−/−^Apoe^−/−^ mice over a 28-day time course. Thus, this study was conducted to investigate whether IKK*ε* serves as a detrimental adaptive mechanism in response to Ang II infusion.

## 2. Materials and Methods

### 2.1. Human Specimens

Human aneurysm samples were collected from the aorta of patients with AAA who were undergoing elective surgery. The control samples were obtained from normal heart transplantation donors who were without an aortic aneurysm (Table 1). All research involving human samples were conducted according to the principles outlined in the Declaration of Helsinki and was approved by the Ethics Committee at Nanjing Medical University. Written informed consent was obtained from the patients and families of the donors.

### 2.2. Ang II-Induced AAA Mouse Model and Treatment

All experiments in this study were performed in accordance with the protocols approved by the Institutional Animal Care and Use Committee of Nanjing Medical University. IKK*ε*^−/−^ and Apoe^−/−^ mice were purchased from the Jackson Laboratory (Bar Harbor, USA) and the Model Animal Research Center of Nanjing University (Nanjing, China), respectively. Apoe^−/−^ IKK*ε*^−/−^mice were generated by crossing IKK*ε*^−/−^ mice with Apoe^−/−^ mice, as reported previously [6]. Alzet model 2004 osmotic minipumps (ALZA Corp, USA) containing 1000 ng/kg/min Ang II (Sigma, USA) or saline were implanted subcutaneously into Apoe^−/−^ IKK*ε*^−/−^ or Apoe^−/−^ mice, respectively, over the course of 28 days, as previously described [10]. A separate group of Apoe^−/−^ mice was orally gavaged with 25 mg/kg of selective IKK*ε* inhibitor, amlexanox (Sigma, St. Louis, USA), or its vehicle (water) every day for 1 week before Ang II infusion, which lasted the duration of the experimental period. After 28 days of Ang II infusion, all mice were sacrificed under anesthesia.

### 2.3. Basic Measurements of Ultrasound Recording for Abdominal Aortas

Mice were anaesthetized with 1.5% isoflurane inhalation and then placed onto a temperature-controlled table. After the hair was removed from the abdomen, an abdominal echography was performed using a Vevo 2100 ultrasound with a 30 MHz transducer applied to the abdominal wall to visualize the abdominal aorta (VisualSonics, Canada). B-mode ultrasound (US) imaging was used to determine the suprarenal abdominal aortic diameter using a real-time microvisualization scan head (RMV 704, Visual Sonics) with a central frequency of 40 MHz.

### 2.4. Measurements of Blood Pressure and Plasma Cholesterol

A noninvasive tail-cuff method was used to measure systolic blood pressure (SBP) using a non-preheating MK-2000ST system (Panlab, Spain). Conscious mice were placed in special mouse holders and acclimated to the device for 10 min before measurement. A minimum of 3 serial measurements was taken, and the average value was calculated. The SBP of each mouse was measured at baseline before Ang II infusion and at 4 weeks after infusion. Four weeks after saline or Ang II infusion, the mice have fasted and blood was collected. Concentrations of plasma total cholesterol were then measured using an automatic biochemistry analyzer (WAKO, USA).

### 2.5. Quantification of Ang II-Induced AAA in Mice

Animals were sacrificed at the end of the interventions. The maximal outer diameter of the suprarenal aorta was measured with Image-Pro Plus software (Media Cybernetics, USA). Aneurysm was defined as an increase in the external width of the suprarenal aorta by at least 50% compared with the vehicle mice, as previously described [11]. The average diameter of the normal suprarenal aorta in control mice is 0.8 mm. Therefore, we set a threshold of 1.2 mm as evidence of aneurysm formation. Based on previous studies [11], aneurysm severity was rated from as follows: none, no aneurysm; Type I, aorta with dilated lumen without thrombus; Type II, aorta with tissue destruction that contains thrombus; Type III, aorta with a pronounced bulbous form that contains thrombus; Type IV, aorta with multiple aneurysms containing thrombus; and Type V, ruptured aorta.

### 2.6. Specimen Preparation and Staining

Abdominal aortic tissues containing AAA were harvested, fixed in 4% paraformaldehyde in phosphate-buffered saline (PBS), and embedded in paraffin for histological analysis. Some aortic tissues were obtained and kept frozen in liquid nitrogen immediately, and then stored at -80°C. Western blotting, immunofluorescence, and measurement of the oxidative radical were performed. Cross sections (5 *μ*m) were prepared and subsequently stained with hematoxylin and eosin (HE) or elastic Van Gieson (EVG) staining according to standard protocol. The elastin integrity was graded as a previously established standard for the elastin degradation score [12].

### 2.7. ELISA Assay

Aorta sections were washed by saline and homogenized aortic homogenates, and cell culture medium was centrifuged. The supernatant was then harvested for ELISA. The levels of cytokines and metalloproteinases were determined using enzyme-linked immunosorbent assay (ELISA) (R&D, Minneapolis, USA) according to the manufacturer's instructions. For this purpose, tumor necrosis factor- (TNF-) *α*, interleukin- (IL-) 6, the chemotactic factor- (MCP-) 1, and matrix metalloproteinase- (MMP-) 2 and MMP9 (R&D, Minneapolis, USA) ELISA kits were utilized.

### 2.8. Gelatin Zymography and In Situ Zymography

MMP activity was measured as previously reported. Briefly, proteins extracted from mice or cell culture medium without boiling were separated by 10% acrylamide-SDS gel containing 0.1% gelatin at 4°C to detect the activities of MMP2 and MMP9. Proteins were denatured in washing buffer for 1 hour, and then the gels were incubated with developing buffer at 37°C for 36 hours. After incubation, the proteins were stained with Coomassie Brilliant Blue and then destained with destaining buffer until clear bands appeared on the blue background. MMP activities were quantified by ImageJ software (Media Cybernetics, USA). For in situ zymography, freshly cut frozen aortic sections (10 *μ*m) were incubated with a fluorogenic gelatin substrate (DQ gelatin; Invitrogen, MA) according to the manufacturer's protocol. The gelatinolytic activity was detected as green fluorescence by a fluorescence microscope (Zeiss Axio Scope A1, Germany).

### 2.9. Immunohistology Staining

Immunohistochemical staining was performed as described previously [13]. Briefly, formaldehyde-fixed paraffin sections (5 *μ*m) were subjected to heat-mediated antigen retrieval. Endogenous peroxidase activity was quenched by treatment with 3% H_2_O_2_ for 10 min at room temperature, blocked with 5% blocking bovine serum albumin solution for 1.5 hours at room temperature, and then incubated with primary antibodies overnight at 4°C. The primary antibodies used were IgG negative control (1 : 200; CST, USA), rabbit anti-IKK*ε* (1 : 200; CST, USA), mouse anti-*α*-smooth muscle actin (*α*-SMA) (1 : 400; Sigma, USA), rabbit anti-cleaved caspase-3 (1 : 200; CST, USA), and rabbit anti-p-ERK1/2 (1 : 200; CST, USA); the sections were then incubated with a MaxVision^TM^ IHC Kit (Fuzhou Maixin Biotech, China). All sections were visualized via the addition of diaminobenzidine (DAB, Vector Laboratories, USA) and counterstained with Mayer's hematoxylin (Histolab Products, USA). The immunohistochemical staining results were quantified by Image-Pro Plus software (Media Cybernetics, USA).

### 2.10. Immunofluorescence Staining

Slides with the frozen aorta sections were air-dried and fixed in 4% paraformaldehyde for 15 minutes, washed 3 times (5 min each time) with PBS, and then blocked with 5% normal goat serum for 60 min at room temperature in a humid chamber. Sections were subsequently incubated overnight at 4°C with the following primary antibodies applied: mouse anti-*α*-SMA (1 : 400), mouse anti-CD68 (1 : 500; Abcam, USA), mouse anti-MCP1 (1 : 500; Santa Cruz Biotechnology, USA), rabbit anti-MMP2 (1 : 500; Abcam, USA), rabbit anti-MMP9 (1 : 500; Abcam, USA), rabbit anti-IL6 antibody (1 : 300; Abcam, USA), and rabbit anti-TNF*α* (1 : 500; Abcam, USA). Sections were washed 3 times (5 min each time) with PBS, Alexa Fluor 488 goat anti-mouse IgG (1 : 500; Invitrogen, USA), and Alexa Fluor 594 goat anti-rabbit IgG (1 : 500; Invitrogen, USA), and the sections were incubated in secondary antibodies for 60 minutes at room temperature in a light-protected humid chamber. The sections were washed 3 times (5 min each time) with PBS and stained with 4,6-diamidino-2-phenylindole (DAPI, 1 : 30, Sigma, USA). Slides incubated with secondary antibodies were used as negative controls.

### 2.11. ROS Analysis

Mice were perfused with cold PBS for 5 min and the abdominal aortas were harvested, embedded in OCT compounds (Tissue-Tek; Sakura Finetek, Japan), and snap-frozen. The frozen segments were cut into 5 *μ*m cryostat sections, which were incubated in 10 *μ*mol/L of dihydroethidium (DHE) (Beyotime Biotechnology, China) at 37°C for 30 min in a humidified chamber and protected from light. Fluorescence was detected using fluorescence microscopy (Zeiss Axio Scope A1, Germany), and the red fluorescence intensity was analyzed using Image-Pro Plus software (Media Cybernetics, USA).

### 2.12. TUNEL Assay

TUNEL staining was conducted using POD, an in situ cell death detection kit (Roche, USA). According to the manufacturer's instructions, in brief, following deparaffinization and rehydration, the sections were incubated with 10 mM protease K for 20 min. The slides were immersed in a TUNEL reaction mixture for 60 min at 37°C in a humidified atmosphere in the dark. The slides were incubated in Converter-POD for 30 min and then rinsed with PBS, and the signal was amplified with horseradish peroxidase-conjugated streptavidin. All sections were counterstained with Mayer's hematoxylin for 30 s.

### 2.13. Cell Isolated, Culture, and Ang II Treatment

Primary VSMCs were isolated from mouse aortas using a standard protocol [14]. We used primary mouse aortic VSMC isolated from Apoe^−/−^ and Apoe^−/−^IKK*ε*^−/−^ mice. The primary VSMCs were maintained in Dulbecco's modified Eagle's medium (DMEM) containing 20% fetal bovine serum (FBS) at 37°C in a humidified atmosphere of 5% CO_2_ and 95% air. The cells at passages 3 to 7 were used for subsequent experiments. Cells were seeded in multiwell plates at a density of 1.0 × 10^4^ cells/cm^2^. Before Ang II (1 *μ*mol/L) treatment for 24 hours, cells were serum-starved in DMEM for 24 h.

### 2.14. Western Blot Analysis

Total protein was extracted from whole aortas or by lysing cells in radioimmunoprecipitation assay (Beyotime Institute of Biotechnology, China). Western blots were performed using various antibodies. Briefly, equal amounts of protein samples were separated by 10% SDS-PAGE, transferred onto polyvinylidene difluoride (PVDF) membranes, and blocked in 5% milk protein. The membranes were incubated with appropriate primary antibodies for either GAPDH (1 : 1000; CST, USA), IKK*ε* (1 : 1000; CST, USA), ERK (1 : 1000; CST, USA), p-ERK1/2 (1 : 1000; CST, USA), P38 (1 : 1000; CST, USA), p-P38 (1 : 1000; CST, USA), JNK (1 : 000; CST, USA), p-JNK (1 : 1000; CST, USA), AKT (1 : 000; CST, USA), and p-AKT (1 : 000; CST, USA) at 4°C overnight. Subsequently, the membranes were incubated with peroxidase-conjugated secondary antibodies for either anti-rabbit or mouse HRP-conjugated IgG (1 : 5000; CST, USA) at room temperature for 1 h. The blots were visualized with enhanced chemiluminescence detection reagent (Applygen Technologies, China). GAPDH expression was used as an internal control. Bands were quantified by densitometry using Gel-Pro Analyzer 4.0 software (Media Cybernetics, USA).

### 2.15. Statistical Analysis

All data are expressed as the mean ± standard errors of the mean (SEM). A Mann–Whitney test was used to examine differences between the two groups. A chi-square test was applied to comparisons of AAA incidence and severity, and a Kaplan-Meier curve test was used for survival curves. Comparisons among three or more groups were performed using one-way ANOVA and post hoc analysis with Bonferroni correction. Data were calculated using GraphPad Prism software, version 6.0 (GraphPad Software Inc., USA). Differences with *P* < 0.05 were considered statistically significant.

## 3. Results

### 3.1. Expression of IKK*ε* Is Upregulated in AAA Tissues from Human and Apoe^−/−^ Mice

**Figure 1:**
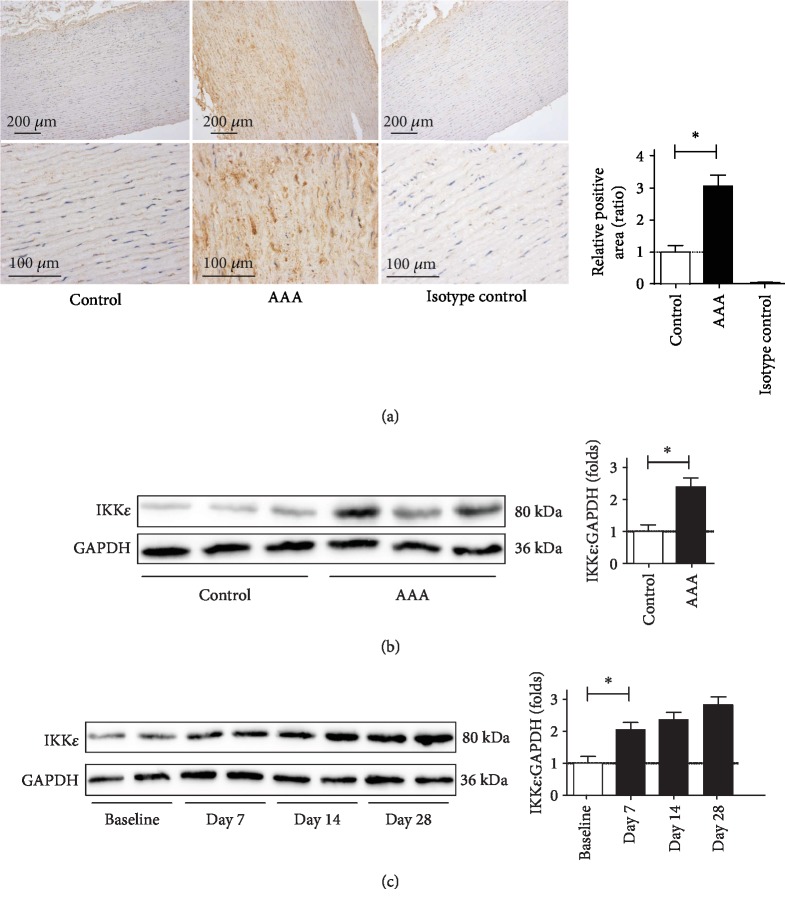
The expression of IKK*ε* is increased in the aortic aneurysm. (a) Representative microscopic photos of immunohistochemical staining for IKK*ε* expression in the control and AAAs. (b) Immunoblots to analyze the expression of IKK*ε* in control human nonaneurysmal aortas vs. AAAs, *n* = 4‐5, respectively. (c) Time course of IKK*ε* expression after infusion with Ang II for 7, 14, and 28 days. Western blot analysis of IKK*ε* protein level in different groups, *n* = 4‐5, respectively.

Previous studies have shown that IKK*ε* plays an important role in regulating cardiovascular disease [6, 7]. Therefore, we assessed IKK*ε* expression in abdominal aortic tissues taken from AAA patients and nonaneurysmal controls. IKK*ε* protein levels were high in the media and adventitia (Figure 1(a)). Western blot analyses demonstrated elevations of IKK*ε* in human AAA tissues (Figure 1(b)) which demonstrates that IKK*ε* is expressed in the aorta and that its expression is increased in AAA. To determine whether the expression of IKK*ε* is altered during AAA formation and progression, we next induced aneurysm in Apoe^−/−^ mice by Ang II infusion (1000 ng/kg/min). We then examined the expressions of IKK*ε* in the aortas of Ang II-infused mice. We found that Ang II infusion for 7 days leads to a significant increase in the expression of IKK*ε* in aorta tissue in this model (Figure 1(c)), and the uptrends remain to the end of infusion. Together, these results suggest that IKK*ε* is significantly increased during AAA formation and may play an important role in this process.

### 3.2. IKK*ε* Deficiency Protects against Ang II-Induced Aortic Aneurysm Formation

**Figure 2:**
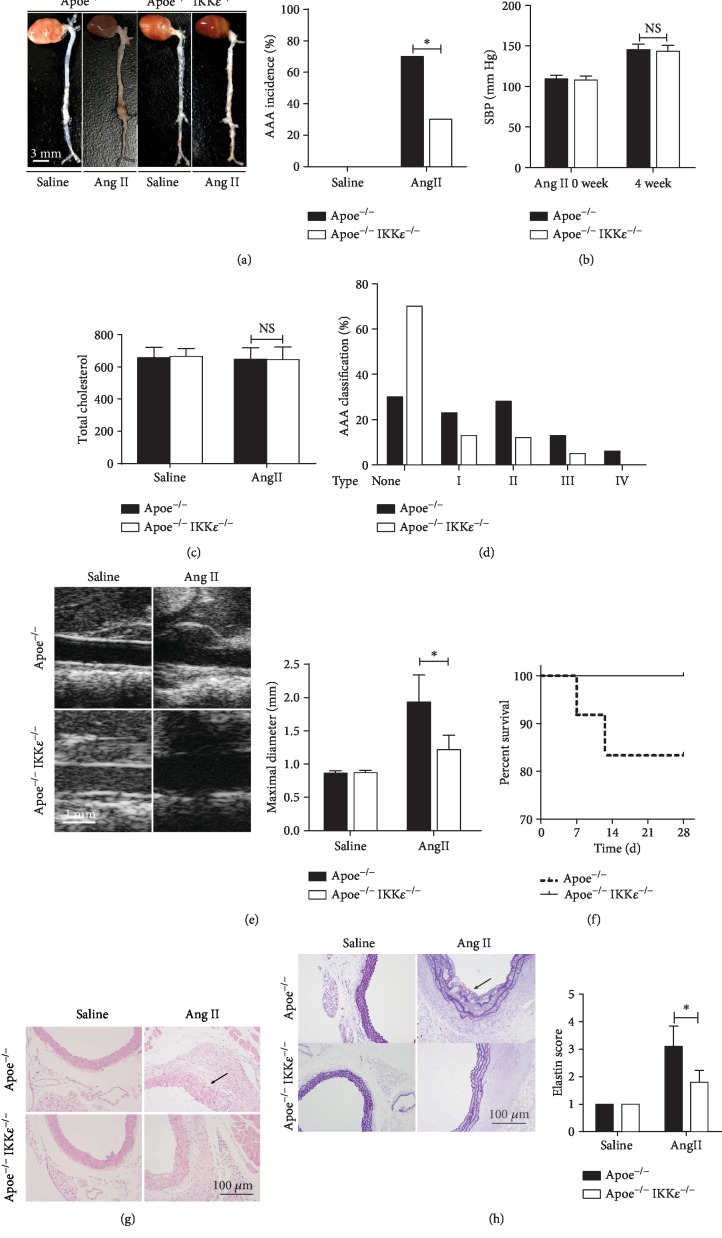
IKK*ε* deficiency protected from Ang II-induced AAA formation in Apoe^−/−^ mice. (a) Representative images of whole aortas from Apoe^−/−^ versus Apoe^−/−^IKK*ε*^−/−^ mice were treated with Ang II or saline for 4 weeks. The bar graph shows the incidences of abdominal aortic aneurysm AAA incidence, *n* = 13‐15, respectively. (b) The systolic blood pressure, *n* = 5‐6, respectively. (c) The total cholesterol levels from plasma, *n* = 5‐6, respectively. NS indicates no significance. (d) Classification severity of aortic aneurysm, *n* = 12‐18 mice per group. ^∗^*P* < 0.001 vs. Apoe^−/−^IKK*ε*^−/−^ mice. (e) Representative images of aortas' ultrasound. The bar graph shows the maximal diameter of the abdominal aorta, *n* = 13‐15, respectively. (f) Kaplan-Meier survival curve in Apoe^−/−^ versus Apoe^−/−^IKK*ε*^−/−^ mice following 4 weeks of Ang II infusion. (g) Representative HE staining of the aortic wall in Apoe^−/−^ versus Apoe^−/−^IKK*ε*^−/−^ mice following 4 weeks of saline or Ang II infusion. (h) Representative EVG staining of the aortic wall in Apoe^−/−^ versus Apoe^−/−^IKK*ε*^−/−^ mice following 4 weeks of saline or Ang II infusion. Quantification is shown in the right panel, *n* = 4‐5, respectively.^∗^*P* < 0.001 vs. Apoe^−/−^IKK*ε*^−/−^ mice.

In view of the increased observation of IKK*ε* expression in AAA, we hypothesized that IKK*ε* deficiency could attenuate AAA formation. IKK*ε* knockout did not alter the general morphology of the aorta in Apoe^−/−^ mice (Figure 2(a)). Age-matched male Apoe^−/−^ and Apoe^−/−^ IKK*ε*^−/−^ mice were subjected to saline or Ang II (1000 ng/kg/min) for 28 days. The saline infusion did not cause any aneurysm formation (Figure 2(a)), and more than 70% of Apoe^−/−^ mice developed AAA, while only 30% of Apoe^−/−^ IKK*ε*^−/−^ mice developed such lesions (Figure 2(a)). In addition, after 28 days of Ang II infusion, although systolic blood was significantly increased compared to the baseline, there was no significant difference between Apoe^−/−^ mice and Apoe^−/−^ IKK*ε*^−/−^ mice (Figure 2(b)). Similarly, there was no difference in total cholesterol levels observed in both saline and Ang II groups in Apoe^−/−^ and Apoe^−/−^ IKK*ε*^−/−^ mice after 4 weeks of Ang II infusion (Figure 2(c)), demonstrating that inactivation of IKK*ε* does not affect systemic metabolism in this mouse model. We further classified the AAA formed in Apoe^−/−^ and Apoe^−/−^ IKK*ε*^−/−^ mice based on the AAA severity suggested by the extensive use of previous classification systems in this experimental model, and the results are shown in (Figure 2(d)). Notably, the AAA phenotype of mice after Ang II infusion demonstrated by ultrasound imaging (Figure 2(e)) that Apoe^−/−^ and Apoe^−/−^ IKK*ε*^−/−^ mice with Ang II infusion significantly increased suprarenal aorta diameter. We demonstrated that the mean maximal aorta diameter of the Apoe^−/−^ IKK*ε*^−/−^ mice treated with Ang II was significantly less than the mean maximum aorta diameter of the Apoe^−/−^ mice treated with Ang II. Interestingly, approximately 18% of Apoe^−/−^ mice infused with Ang II died within the first 14 days of the 4-week Ang II stimulation, whereas none of the Apoe^−/−^ IKK*ε*^−/−^ mice died during the entire experiment (Figure 2(f)). It is reported that artery wall remodeling is a hallmark of AAA formation and progression. HE staining was performed on aortic sections from the suprarenal aorta, which specifically demonstrates marked enlargement of the internal diameter, destruction of the media, and marked thickening of the aortic wall in Ang II-treated Apoe^−/−^ mice (Figure 2(g)). However, IKK*ε* deficiency dramatically reversed this damage, and normal morphology of suprarenal aorta was exhibited in saline-treated Apoe^−/−^ and Apoe^−/−^ IKK*ε*^−/−^ mice. Characteristic vessel wall remodeling in aneurysms was demonstrated by EVG staining, and continuous and wavy elastic lamellae could be found in saline-treated Apoe^−/−^ and Apoe^−/−^ IKK*ε*^−/−^ mice (Figure 2(h)), as well as degradation of elastin filaments in both in Ang II-treated mice. However, compared with the Apoe^−/−^ mice (Figure 2(h)), the degree of elastin filament degradation was lower in Apoe^−/−^ IKK*ε*^−/−^ mice (Figure 2(h)). These findings suggest that Apoe deficiency may attenuate AAA formation and progression by protecting from elastin degradation and by promoting and maintaining the normal aortic structure.

### 3.3. IKK*ε* Deficiency Attenuated MMP Expression and Activation in Ang II-Induced AAA

**Figure 3:**
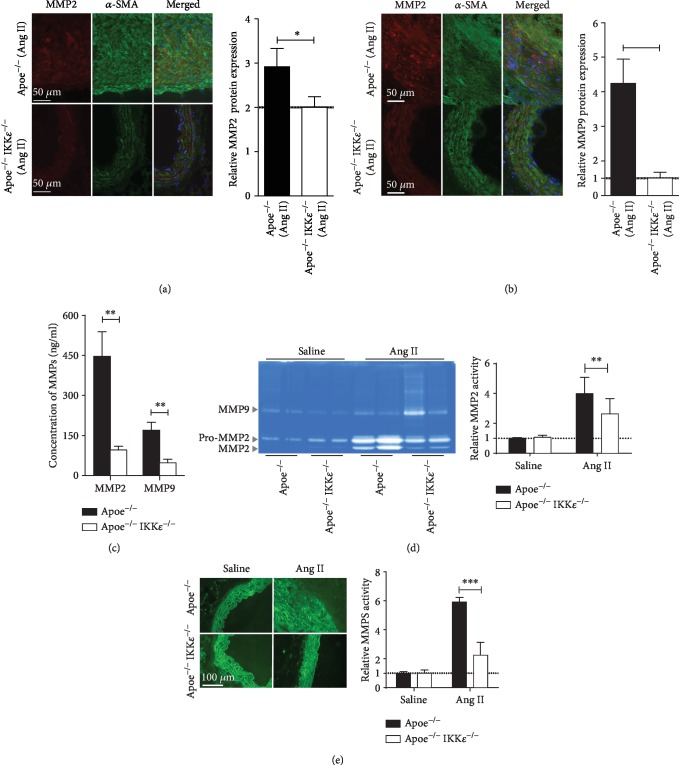
IKK*ε* deficiency attenuates MMP production. Double staining for (a) MMP 2 red and *α*-SMA green or (b) MMP9 red and *α*-SMA green in aortic sections from mice in the indicated groups after 4 weeks of infusion. Nuclei were counterstained with DAPI blue. Quantification is shown in the right panel, *n* = 4‐5, respectively. (c) MMP expression in abdominal aortic protein lysates by ELISA, *n* = 4‐5, respectively. (d) MMP activity in abdominal aortic protein lysates by gelatin zymography. Quantification is shown in the right panel, *n* = 4‐5, respectively. (e) The fresh-frozen sections were analyzed by in situ zymography with DQ gelatin to detect the gelatinolytic activity of MMPs. Quantification is shown in the right panel. *n* = 4‐5, respectively. ^∗^*P* < 0.05 vs. Apoe^−/−^IKK*ε*^−/−^ mice. ^∗∗^*P* < 0.01 vs. Apoe^−/−^IKK*ε*^−/−^ mice. ^∗∗∗^*P* < 0.001 vs. Apoe^−/−^IKK*ε*^−/−^ mice.

MMP2 and MMP9 have been shown to contribute to damage to the integrity of the arterial wall and the progress of AAA formation in experimental studies [15]. The results showed that MMP2 and MMP9 were widely screened by immunofluorescence in Apoe^−/−^ mice injected with Ang II compared with Apoe^−/−^ IKK*ε*^−/−^ mice injected with Ang II (Figures 3(a) and 3(b)). We obtained the confirmed results by ELISA. The MMP expression was significantly reduced in Apoe^−/−^IKK*ε*^−/−^ mice (Figure 3(c)) compared with Apoe^−/−^ mice after Ang II infusion. Consistent with the increase in MMP expression, to determine whether altered MMP levels translate into proteolytic activity, MMP activity in aortic homogenates from Apoe^−/−^ and Apoe^−/−^IKK*ε*^−/−^ mice was evaluated by zymography. The gelatin zymogram from the aorta of Apoe^−/−^ showed a significant increase in MMP2 activation. In contrast, Ang II-treated aorta from Apoe^−/−^IKK*ε*^−/−^ mice showed a significant reduction in MMP2 (Figure 3(d)). We obtained the confirmed results by in situ zymography. The activity of MMP (green fluorescence) was significantly reduced in Apoe^−/−^IKK*ε*^−/−^ mice (Figure 3(e)) compared with Apoe^−/−^ mice after Ang II infusion. The activity of Apoe^−/−^ and Apoe^−/−^IKK*ε*^−/−^ mice was negligible after saline infusion. The less severe elastin degradation of the aortic aneurysm aorta may be attenuated by MMP activity. In summary, these studies suggested that following Ang II infusion, the aortas of Apoe^−/−^IKK*ε*^−/−^mice were characterized by reduced MMP expression associated with depressed MMP activity.

### 3.4. IKK*ε* Deficiency Prevents Aortic Inflammatory Cell Infiltration in Ang II-Induced AAA

**Figure 4:**
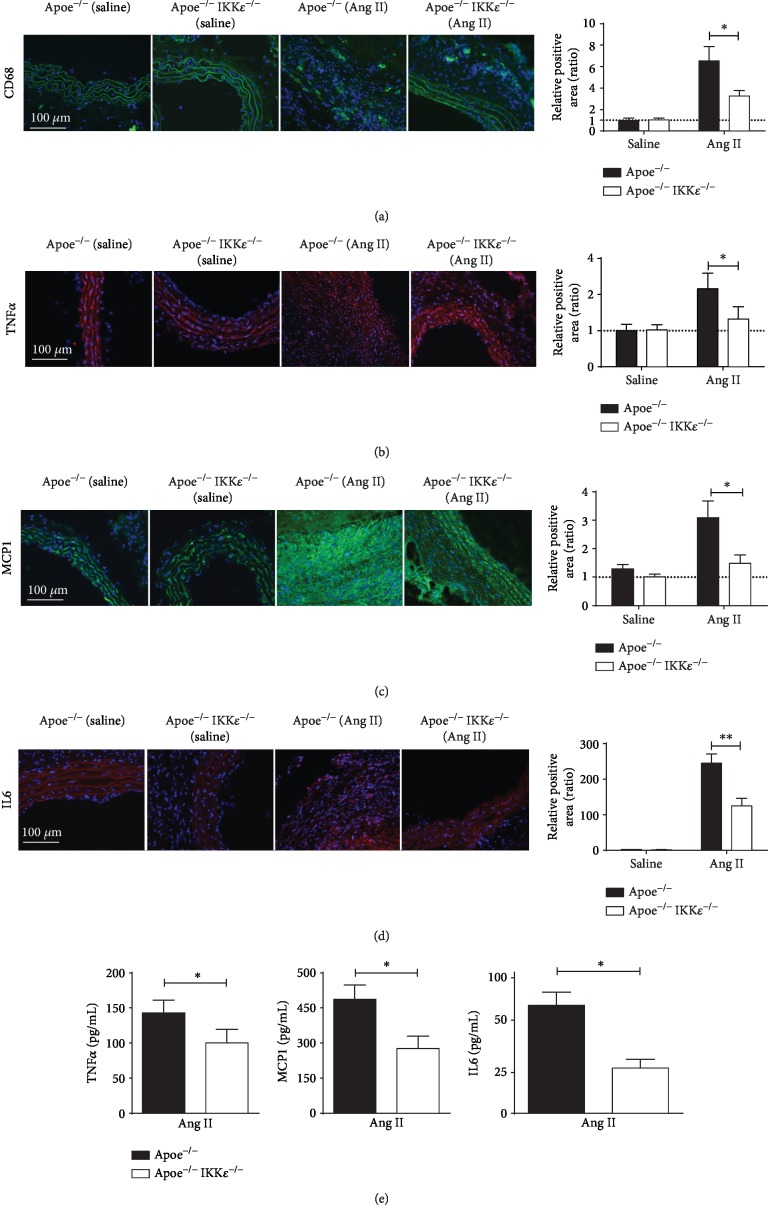
IKK*ε* deficiency diminished a proinflammatory response. Representative images of immunofluorescence staining for (a) CD68, (b) TNF*α*, (c) MCP1, and (d) IL6 of the aortic wall in Apoe^−/−^ versus Apoe^−/−^IKK*ε*^−/−^ mice following 28 days of saline or Ang II infusion *n* = 4‐5, respectively. Quantification shown in the right panels. (e) TNF*α*, MCP1, and IL6 expression in aortic tissue samples detected by ELISA. ^∗^*P* < 0.05 vs. Apoe^−/−^IKK*ε*^−/−^ mice, ^∗∗^*P* < 0.01 vs. Apoe^−/−^IKK*ε*^−/−^ mice.

To further evaluate the effects of IKK*ε* deficiency on the macrophage-mediated inflammation present during the aneurysm progression, we first performed immunofluorescence studies to detect macrophages in sections of the suprarenal aorta of Apoe^−/−^ mice and Apoe^−/−^IKK*ε*^−/−^ controls at the 28-day time point. However, as expected and as assessed by CD68 (macrophage) staining, Ang II infusion led to the accumulation of inflammatory cells within the aortas of mice. A significant decrease was found in Apoe^−/−^IKK*ε*^−/−^ mice infused with Ang II as compared with Apoe^−/−^mice infused with Ang II (Figure 4(a)), and IKK*ε* deficiency significantly diminished Ang II-induced inflammatory response; studies were performed to assess the expression of several key inflammatory molecules implicated in AAA [12, 13]. Consistent with enhanced inflammatory immunoreactivity, immunofluorescence staining demonstrated that the numbers of proinflammatory cytokines TNF*α*, IL6, and the MCP1 were dramatically blunted in the Apoe^−/−^IKK*ε*^−/−^ mice relative to Apoe^−/−^ mice after Ang II infusion for 28 days (Figures 4(b)–4(d)). A few inflammatory cells and other cytokines were found in the suprarenal aortic wall of saline-infused mice. We obtained the confirmed results by ELISA. The proinflammatory cytokines were significantly reduced in Apoe^−/−^IKK*ε*^−/−^ mice (Figure 4(e)) compared with Apoe^−/−^ mice after Ang II infusion. Overall, these results suggest that IKK*ε* deficiency ameliorated inflammatory actions in the vascular wall that may protect mice from aneurysm development.

### 3.5. IKK*ε* Deficiency Attenuated Aortic ROS in Ang II-Induced AAA

**Figure 5:**
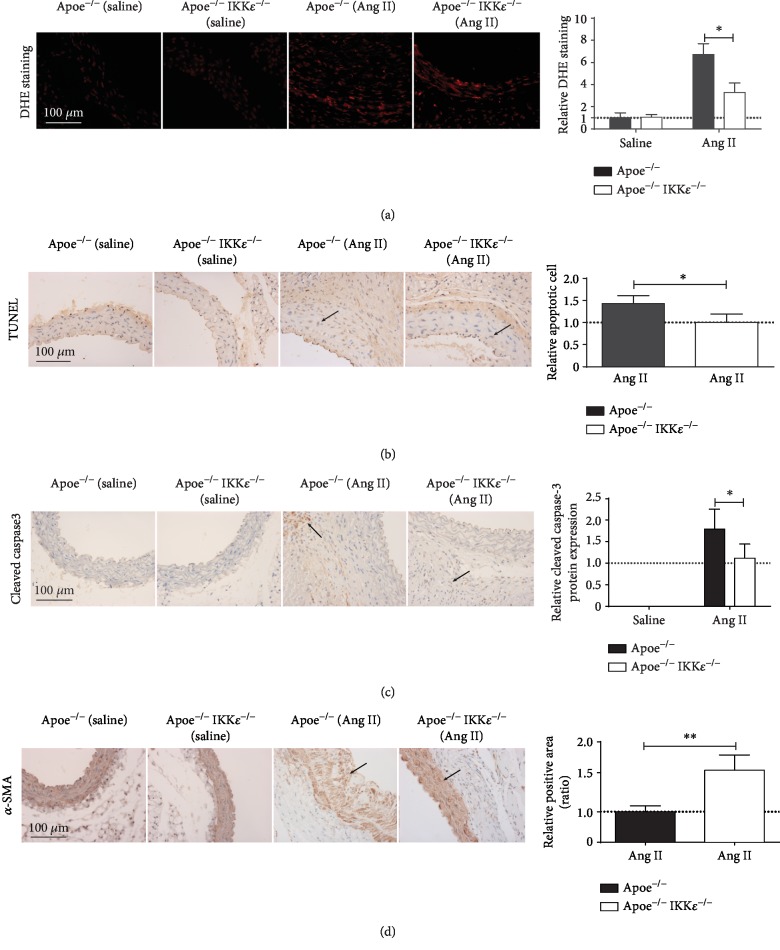
IKK*ε* deficiency diminished a ROS production and VSMC loss. (a) Representative in situ DHE staining of aortas from Apoe^−/−^ versus Apoe^−/−^IKK*ε*^−/−^ mice infused with saline or Ang II for 28 days. *n* = 4‐5, respectively, Quantification shown in the right panels. (b) TUNEL staining. (c) cleaved caspase-3 staining in aortic sections at 28 days post infusion. *n* = 4‐5, respectively. Quantification shown in the right panels. (d) *α*-SMA staining of aneurysmal aortic sections from mice. *n* = 4‐5, respectively. Quantification shown in the right panels. ^∗^*P* < 0.05 vs. Apoe^−/−^IKK*ε*^−/−^ mice, ^∗∗^*P* < 0.01 vs. Apoe^−/−^IKK*ε*^−/−^ mice.

Previous research from animal and human studies in the pathogenesis of AAA indicates that ROS is a key mediator [16]. We incubated abdominal aortic sections from Ang II or saline-infused Apoe^−/−^ and Apoe^−/−^IKK*ε*^−/−^ mice with DHE, which, in the presence of superoxide, forms the highly fluorescent molecule oxyethidium. Following 28 days of Ang II infusion, we observed strong DHE staining in the media and aneurysms in both genotypes of mice. In comparison, the staining intensity of Apoe^−/−^ mice was much stronger than Apoe^−/−^IKK*ε*^−/−^ mice (Figure 5(a)). As expected, in saline-infused mice, both Apoe^−/−^ and Apoe^−/−^IKK*ε*^−/−^ aorta demonstrated minimal evidence of ROS (Figure 5(a)). These findings demonstrate that lack of IKK*ε* reduces ROS accumulation in the Ang II-infused aneurysmal aorta.

### 3.6. IKK*ε* Deficiency Attenuated AAA Cell Apoptosis in Ang II-Infused Mouse Aorta

Previous research has shown that increased inflammation and elevated ROS promote exaggerated vascular smooth muscle cell (VSMC) apoptosis [17]. Therefore, we evaluated that loss of IKK*ε* may attenuate apoptosis in the aneurysmal aorta. After 28 days of Ang II infusion, IKK*ε* deficiency attenuated TUNEL-positive cells in tissue sections (Figure 5(b)). Similar effects were observed by immunostaining with cleaved caspase-3 antibody, which revealed fewer apoptotic cells in the aortic media from Apoe^−/−^ IKK*ε*^−/−^ than Apoe^−/−^mice (Figure 5(c)). In the same result, IKK*ε* knockout decreased the VSMC loss which labeled by the *α*-SMA (Figure 5(d)). In the saline-infused animals, we observed minimal apoptotic cells in both Apoe^−/−^ and Apoe^−/−^ IKK*ε*^−/−^ mice. These findings demonstrate that Apoe^−/−^IKK*ε*^−/−^ mice attenuated the loss of VSMC compared with Apoe^−/−^ mice at 28 days after Ang II infusion.

### 3.7. IKK*ε* Regulated Ang II-Activated ERK1/2 Signaling Pathways

**Figure 6:**
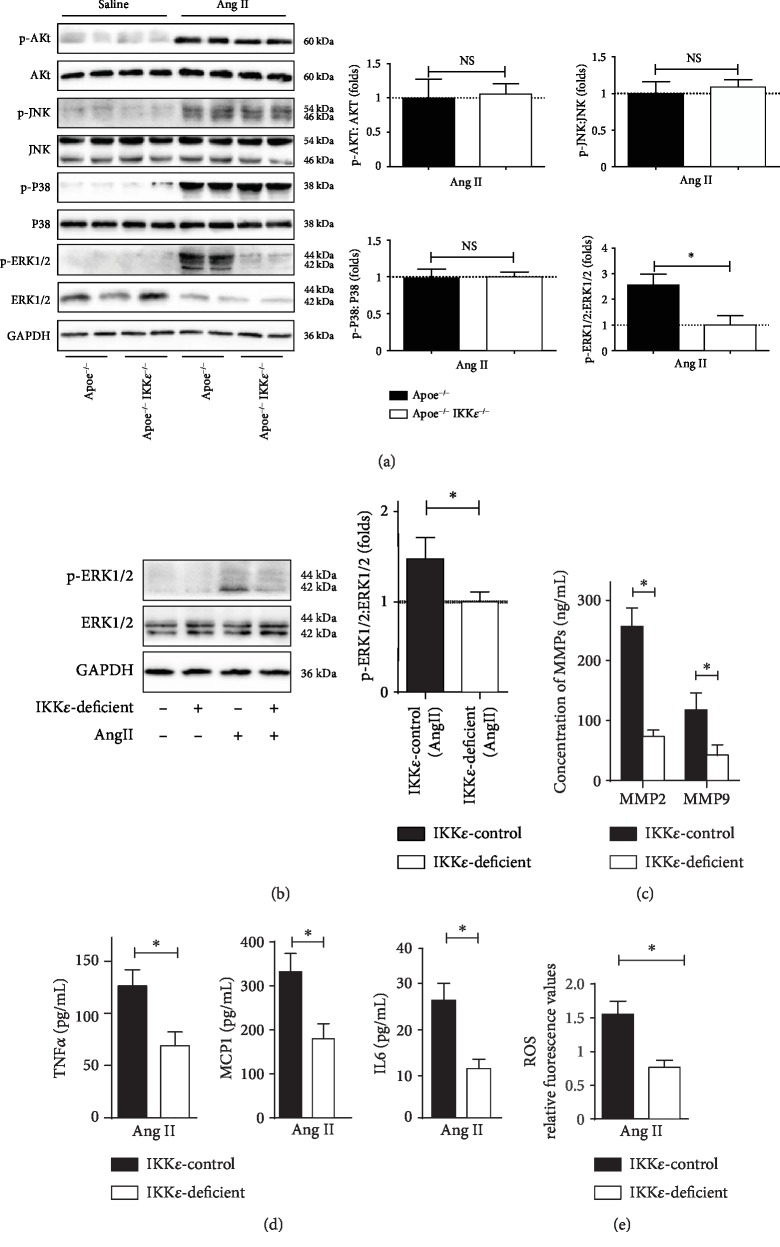
IKK*ε* deficiency blocked phosphorylation of ERK1/2. (a) Western blot analysis for the total and phosphorylated protein levels of AKT, JNK, P38, and ERK1/2 in aortic tissues from mice in the indicated groups at 28 days of infusion. Quantification shown in the right panels, *n* = 4‐5, respectively. ^∗^*P* < 0.05 vs. Apoe^−/−^IKK*ε*^−/−^ mice. (b) Total and phosphorylated protein levels of ERK1/2 in primary VSMC in the indicated groups. Quantification shown in the right panels. (c) MMP expression in primary VSMC in the indicated groups detected by ELISA. (d) TNF*α*, MCP1, and IL6 expressions in primary VSMC in the indicated groups detected by ELISA. (e) Analysis of ROS generation in primary VSMC in the indicated groups by flow cytometer, *n* = 4‐5, respectively.^∗^*P* < 0.05 vs. IKK*ε*-deficient.

This study is an effort to glean mechanistic insights into the underlying cause of AAA pathology, considering the involvement of ERK1/2 signaling in AAA [12] and the published studies demonstrating that IKK*ε* is required for the induction of proinflammatory cytokines [18] and ERK1/2 activation [19]. We further hypothesized that IKK*ε* might participate in the development of AAA via activation of the ERK1/2 pathways. As shown in (Figure 6(a)), we observed a profound inhibition of ERK1/2 signaling in IKK*ε*-deficient aortas, as evidenced by marked abolishment of ERK1/2 phosphorylation at 4 weeks after Ang II infusion by western blot analyses, but not, however, phosphorylated AKT, JNK, or P38.

### 3.8. IKK*ε* Deficiency Attenuated Ang II-Induced ERK1/2 Phosphorylation, MMP Expression, Inflammatory Actions, and ROS Accumulation in Primary VSMC

In order to further corroborate our findings garnered from these rodent models, we also validated the blunted activation of IKK*ε* deficiency in primary VSMC in vitro experiments. In vitro experiments also validated the blunted activation of IKK*ε* deficiency on ERK1/2 (Figure 6(b)). We observed that the Ang II treatment induced the expression of MMP2 and MMP9 in cultured VSMC, whereas IKK*ε* deficiency did not exhibit such an upregulation (Figure 6(c)). ELISA assays showed that IKK*ε* deficiency suppressed Ang II-induced inflammatory cytokines TNF*α*, MCP1, and IL6 (Figure 6(d)). Moreover, we next assessed ROS generation and we observed Ang II-induced ROS productions are inhibited by IKK*ε* deficiency (Figure 6(e)). In summary, we confirmed the protective mechanistic of IKK*ε* deficiency on Ang II-induced activation at least through the ERK1/2 signaling pathways. These observations prompted us to hypothesize that IKK*ε* may play a vital role in AAA formation by ERK1/2 signaling pathway activation.

### 3.9. Pharmacological Inhibition of IKK*ε* Mitigates Ang II-Induced AAA Formation in Apoe-Deficient Mice

**Figure 7:**
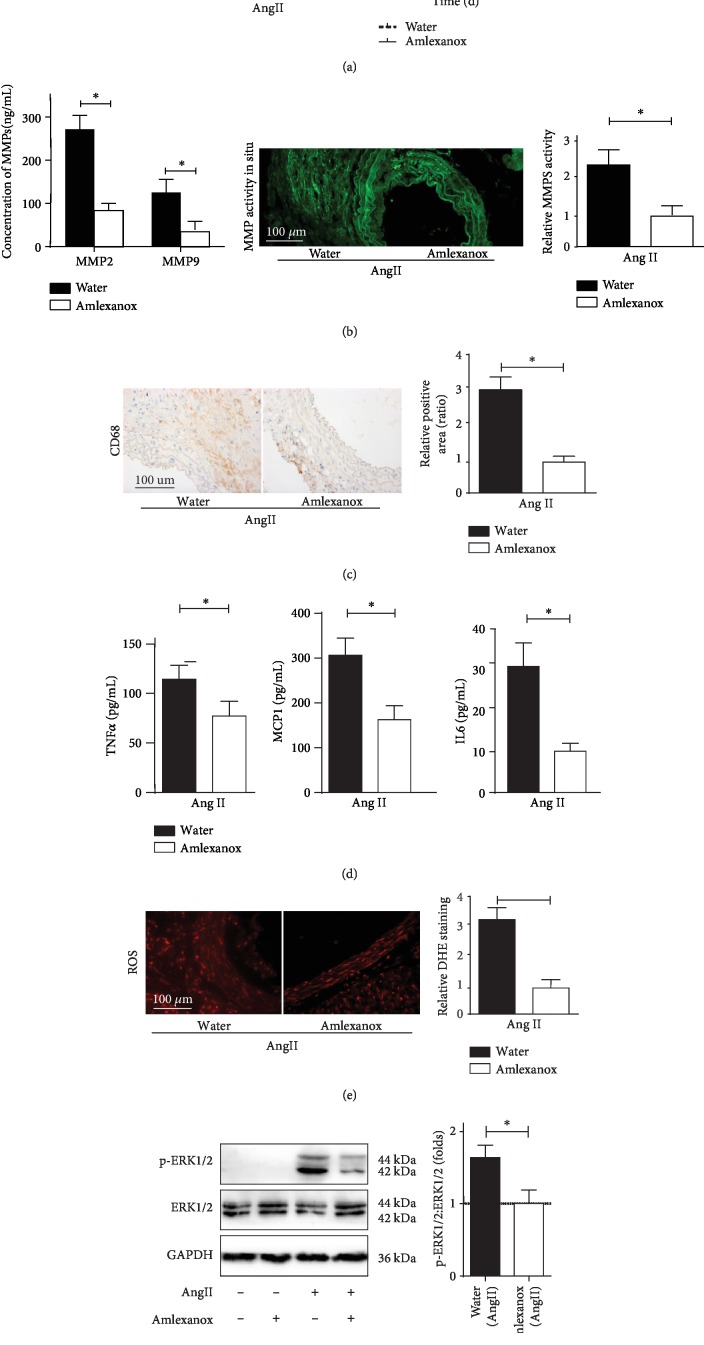
IKK*ε* inhibitor amlexanox treatment reduces Ang II-induced AAA formation in Apoe^−/−^ mice. (a) AAA incidence and Kaplan-Meier survival curve following 4 weeks of Ang II infusion treated with water or amlexanox in Apoe^−/−^mice. *n* = 8‐9 mice per group. (b) MMP expression and activity in Apoe^−/−^ mice following 4 weeks of Ang II infusion treated with water or amlexanox, *n* = 4‐5, respectively. (c) Macrophage infiltration in Apoe^−/−^ mice following 4 weeks of Ang II infusion treated with water or amlexanox, *n* = 4‐5, respectively. (d) TNF*α*, MCP1, and IL6 expression in aortic tissue samples detected by ELISA, *n* = 4‐5, respectively. ^∗^*P* < 0.01 vs. amlexanox treatment mice. (e) Analysis of treated with water or amlexanox control on ROS production, *n* = 4‐5, respectively. Quantification shown in the right panels. (f) Total and phosphorylated protein levels of ERK1/2 in Apoe^−/−^ mice following 4 weeks of Ang II infusion treated with water or amlexanox, *n* = 4‐5, respectively. Quantification shown in the right panels. ^∗^*P* < 0.01 vs. amlexanox treatment mice.

Because IKK*ε* activity has been linked to the pathology of inflammatory diseases [5, 7], a recent study identified an anti-inflammatory drug, amlexanox, as a selective inhibitor of IKK*ε* and an approved small-molecule therapeutic presently used in the clinic to treat aphthous ulcers and in clinical trials for obesity [20]. We assumed that amlexanox administration could efficiently inhibit Ang II-induced AAA in mice; pharmacological inhibition studies were employed using to address this hypothesis. Water or amlexanox (25 mg/kg/d) daily oral gavage administration was initiated 1 week prior to Ang II infusion. Next, all mice were subjected to Ang II infusion with continually received daily oral gavage of vehicle or amlexanox for 28 days. Upon completion of the 4-week Ang II stimulation, in marked agreement with the results from the Apoe^−/−^IKK*ε*^−/−^ mice, amlexanox administration dramatically reduced AAA incidence amd mortality in mice (Figure 7(a)). Moreover, amlexanox administration strongly reversed MMP expression and activation, inflammatory cell accumulation, inflammatory actions, ROS production, and ERK1/2 activation (Figures 7(b)–7(f)). Collectively, these findings indicate that the IKK*ε* inhibitor amlexanox protects Apoe^−/−^ mice from Ang II-induced AAA formation. The use of amlexanox is a potential agent for a nonsurgical approach to the prevention and treatment of AAA.

## 4. Discussion

Previous studies have implicated IKK*ε* in the pathology of a wide array of diseases, including cancer, inflammatory, and metabolic diseases [4, 8, 18]. Many studies indicate a critical role of IKK*ε* in the pathophysiology of inflammatory diseases; it has been reported that inflammation appeared to be reduced in IKK*ε* KO mice [19, 21], and our previous study indicated that deficiency of IKK*ε* inhibits inflammation and induces cardiac protection [7]. However, the direct effects of IKK*ε* on AAA development are still unclear. Therefore, we examined the role of IKK*ε* in AAA using an Ang II-infused Apoe^−/−^ animal model.

Our results support a pathogenic role of IKK*ε* in AAA pathogenesis. We found that IKK*ε* expression is strengthened in both human and mouse AAA tissues. IKK*ε* ablation reduced inflammatory cytokine expression, MMP activity, and macrophage infiltration and inhibited ROS production, leading to forestalling abdominal aortic aneurysm formation. The pharmacological study also confirms the effect. Mechanistically, our results indicate the molecular events underlying IKK*ε*'s involvement in AAA formation depend at least in part on the accelerated phosphorylation of ERK1/2, suggesting that IKK*ε* is a potential novel therapeutic target for AAA progression.

Proteolytic degradation within the vessel wall mediated largely by dysregulation of MMP production and activity is an additional major mechanism in AAA initiation and progression [22, 23]. MMPs are a major cause that degrades the structural components of the ECM, and the formation and progression of AAA are accompanied by extracellular matrix degeneration. Previous studies have shown that MMP activity is elevated in AAA. Among the MMPs, especially MMP2 and MMP9, are considered the major proteases in the aortic wall destruction process [24]. In this study, IKK*ε* deficiency significantly inhibited expressions and activities of MMPs. However MMP9 activity is elevated in IKK*ε*-deficient mice, we need to further study the specific mechanism.

Increased infiltration of macrophages into the aneurysmal aortic wall is a hallmark in the pathogenesis of AAAs and has been demonstrated to be the dominant role of inflammatory response found in human and Ang II-induced AAA. It is well established that macrophages secrete proinflammatory chemokines and MMPs, such as TNF*α*, IL6, and MCP1, MMPs, which contribute to macrophage infiltration as an inflammatory circuit. Consistent with our data, IKK*ε* deficiency can downregulate macrophage infiltration in animal disease models [19, 25], suggesting that IKK*ε* exacerbates vascular infiltration of macrophages in Ang II-induced AAA [26]. In other models of inflammatory lesions, IKK*ε* deficiency has been shown to repress proinflammatory gene expression [19]. IKK*ε* deficiency decreased TNF*α* and MCP1 expressions in the white adipose tissue of mice fed with a high-fat diet [25]. Similarly, expression of several chemokines, most notably TNF*α*, IL6, and MCP1, was also lower in the IKK*ε*-deficient mice, leading to reduced lesion formation [5]. Consistent with our data, we confirmed that IKK*ε* deficiency blocked the expression of the proinflammatory cytokines TNF*α*, IL6, and MCP1 in Ang II-induced AAAs. These data are consistent with previous studies showing that Ang II-induced AAAs of IKK*ε*-deficient mice exhibit reduced expression of proinflammatory factors.

Reports from recent clinical and experimental investigations suggest that ROS are involved in the pathological process in AAA [16]. Oxidative stress is closely associated with inflammation and MMP activation [16, 27], and evidence suggests that oxidative stress induces inflammation, and in turn, proinflammatory cytokines may activate macrophages to increase intracellular ROS [28], although the reciprocation between these two processes is poorly known. In the present study, Ang II-induced AAA in Apoe^−/−^ mice was associated with oxidative stress. A decrease in the production of ROS was found in the lesion site in aortas from Apoe^−/−^IKK*ε*^−/−^ mice as compared with Apoe^−/−^ mice. The current study established the dual role of IKK*ε* in oxidative stress and inflammatory response that occurs during Ang II-induced AAA formation.

VSMC-derived MMP2 promotes degradation of collagen and elastin, contributing to AAA formation [15]. Additionally, ROS have also been shown to activate MMP2 directly [29]. Therefore, the significantly reduced expression and activity of MMP2 may be explained by reduced ROS production. The reduced MMP9 expression and activity may be associated with reduced aortic mural macrophage infiltration, considering macrophages are the major sources of MMP9 [15]. Alternatively, the expression of IKK*ε* may have a direct effect on the expression of MMP2 and MMP9. IKK*ε* knockdown is involved in the decreased MMP2 and MMP9 production in glioma cells [30]. Consistent with this previous study, we suppose that the variance of IKK*ε*'s effectiveness on the oxidative stress and inflammatory response may contribute to the difference between MMP2 and MMP9 gene expression and activity.

VSMCs provide structural support for blood vessels and produce collagen and elastin. Arterial wall medial VSMC apoptosis is an important hallmark of AAA. In addition to regulating the degradation and remodeling of the extracellular matrix (ECM) by upregulating proteolytic enzymes such as MMPs [15], ROS have been reported to promote VSMC apoptosis [31]. Consistent with this previous study, we demonstrated that IKK*ε* deficiency reduced aortic VSMC apoptosis and reduced VSMC loss in AAA lesions. Taken together, IKK*ε* deficiency might play an important role in ameliorative VSMC depletion during Ang II-induced AAA formation.

For the downstream signaling pathways, we investigated the mechanistic basis for the pernicious role of IKK*ε* in AAA formation by examining multiple signaling pathways known to be involved in Ang II-induced AAA formation. Interestingly, our data show that IKK*ε* deficiency prevented the phosphorylation of ERK1/2 in aortas of Ang II-mediated Apoe^−/−^mice, but not AKT, JNK, and P38. Previous research showed that the phosphorylation of ERK1/2 in some malignant behavior of tumor cells relies on IKK*ε* [19]. Another important finding of our study was that progression of the lesion in Ang II-induced AAA in Apoe^−/−^ mice was also blunted by pretreatment of amlexanox, a newly identified chemical inhibitor of IKK*ε*, which was shown to produce many of these effects through its anti-inflammatory properties [20]. Amlexanox seems to be a relatively safe drug with a long history of use in patients [20]. As such, there might be an interesting opportunity to repurpose this drug for AAA. Nevertheless, the precise mechanisms by which amlexanox produces these beneficial effects in AAA models have not yet been completely elucidated.

The current study has a number of limitations. First, one of the most widely used animal models is the use of Ang II infusion modeling, which results in many features similar to the human disease [10], but it does not copy the exact pathologic conditions in human AAAs. Further investigations need to use another widely used CaCl_2_-induced model to examine the effects of IKK*ε* deficiency on AAA progression. Second, several other studies assessing the effects of IKK*ε* deficiency in the inflammatory diseases model were associated with a reduction in macrophage infiltration [19]. However, given the central importance in vascular structure and function, VSMC is the most researched cause of the mechanistic insights involved in AAA formation [32], and these results do not provide definitive proof of the importance of macrophages or VSMC. Therefore, to further investigate the exact role of IKK*ε* in AAA pathology, tissue-specific IKK*ε*-deficient mice will be used in the future.

## 5. Conclusion

To the best of our knowledge, we are the first to demonstrate a crucial role of IKK*ε* in Ang II-induced experimental AAA in mice. Attenuated aneurysmal formation in Apoe^−/−^ mouse deficiency in IKK*ε* is associated with reduced inflammatory macrophage infiltration, MMP activity, ROS production, and VSMC apoptosis. We also demonstrated that low-dose IKK*ε* inhibitors prevented these effects in vivo. These findings not only further highlight IKK*ε*'s involvement in AAA pathophysiology but also suggest that IKK*ε* inhibitors might be a new therapeutic target for the treatment of AAA formation. Further investigations into other animal models of AAA are needed to verify the clinical use of IKK*ε* inhibitors as a promising nonsurgical therapeutic option for AAA treatment.

## Figures and Tables

**Table 1:** Patient characteristics.

Characteristics	Control (*n* = 11)	AAA (*n* = 11)
Age (years)	62.3 ± 4.3	63.8 ± 5.7
Male sex	7 (63%)	8 (72%)
History of smoking	4 (36%)	8 (72%)
Hypertension	7 (63%)	9 (81%)
Diabetes mellitus	4 (36%)	3 (27%)
Stroke	5 (45%)	2 (18%)
Aneurysm diameter at sample site (cm)	N/A	6.3 ± 1.7

AAA: abdominal aortic aneurysm, N/A: not applicable. Data are expressed as number and proportion or mean ± standard deviation.

## Data Availability

The data used to support the findings of this study are available from the corresponding author upon request.
